# Fundamental Concepts and Novel Aspects of Polycystic Ovarian Syndrome: Expert Consensus Resolutions

**DOI:** 10.3389/fendo.2020.00516

**Published:** 2020-08-11

**Authors:** Antonio Aversa, Sandro La Vignera, Rocco Rago, Alessandra Gambineri, Rossella E. Nappi, Aldo E. Calogero, Alberto Ferlin

**Affiliations:** ^1^Department of Experimental and Clinical Medicine, University “Magna Graecia”, Catanzaro, Italy; ^2^Department of Clinical and Experimental Medicine, University of Catania, Catania, Italy; ^3^Physiopathology of Reproduction and Andrology Unit, Sandro Pertini Hospital, Rome, Italy; ^4^Department of Medical and Surgical Science, University Alma Mater Studiorum, Bologna, Italy; ^5^Research Center for Reproductive Medicine, Gynecological Endocrinology and Menopause, IRCCS San Matteo Foundation, University of Pavia, Pavia, Italy; ^6^Department of Clinical and Experimental Sciences, University of Brescia, Brescia, Italy

**Keywords:** PCOS, medical therapy, nutraceuticals, PCOS carriers, male PCOS, consensus

## Abstract

Polycystic ovary syndrome (PCOS) is a very common endocrine and metabolic disorder with the involvement of both genetic and environmental factors. Although much has been clarified on its pathogenesis, diagnosis, clinical manifestations, and therapy, there are still areas of uncertainty. To address fundamental concepts, novel aspects and hypotheses, and future perspectives, including the possible additional benefits of treatment with nutraceuticals, an expert consensus panel formed by endocrinologists and gynecologists was established. After an independent review of the literature, the panel convened electronically on February 3, 2020, and six resolutions were created, debated, and agreed upon discussion, and finally approved in their final form in a consensus livestream meeting held on April 15. The summary of the resolutions are: (1) PCOS is a well-established medical condition that negatively affects reproduction, general health, sexual health, and quality of life; (2) the symptoms and signs of PCOS appear early in life especially in female newborns from PCOS carriers; (3) women with PCOS have significantly increased risk of pregnancy-related complications including gestational diabetes mellitus; (4) a male PCOS equivalent exists, and it may impact on metabolic health and probably on reproduction; (5) the evidence supports that medical therapy for PCOS is effective, rational, and evidence-based; (6) the evidence supports a major research initiative to explore possible benefits of nutraceutical therapy for PCOS. The proposed resolutions may be regarded as points of agreement based on the current scientific evidence available.

## Introduction

Polycystic ovary syndrome (PCOS) is a very common endocrine disorder in women of reproductive age, with a reported prevalence ranging from 6 to 15% (1). Its etiology involves both genetic and environmental factors. Typically, women with PCOS show clinical and biochemical hyperandrogenism, oligoanovulation, and micropolycystic morphology of the ovaries (2). However, PCOS diagnosis relies on specific criteria that differ according to the scientific association that released them (Table 1).

**Table 1:** Criteria for the diagnosis of polycystic ovary syndrome (3).

**NIH/NICHD**	**ESHRE/ASRM 2004**	**Androgen Excess Society 2006**
Includes all of the following criteria:	Includes two of the following criteria:	Includes all of the following criteria:
• Clinical and/or biochemical signs of hyperandrogenism	• Clinical and/or biochemical signs of hyperandrogenism	• Clinical and/or biochemical signs of hyperandrogenism
• Menstrual dysfunction	• Oligo-ovulation or anovulation • Polycystic ovaries	• Ovarian dysfunction and/or polycystic ovaries

*ESHRE/ASRM, European Society for Human Reproduction and Embryology/American Society for Reproductive Medicine; NIH/NICHD, National Institutes of Health/National Institute of Child Health and Human Development*.

The diagnostic criteria do not consider the widely recognized dysmetabolic background of PCOS. Indeed, many patients have impaired insulin action [up to 75% of them are insulin-resistant (IR)], hyperinsulinemia, and overweight/obesity, and this appears to play a crucial role in the pathogenesis of PCOS. Two major PCOS phenotypes can be distinguished: the overweight/obese and the lean one. Their estimated prevalence is ≈80 and ≈20%, respectively (4). Insulin resistance is a fundamental pathogenic component of PCOS, both in lean and overweight–obese patients. The expression of the syndrome differs between lean and overweight–obese PCOS. Worryingly, some patients with the lean phenotype may not show symptoms such as irregular menses or acne (5, 6), and this increases the chance of underdiagnosis or misdiagnosis. Women with PCOS are exposed to metabolic alterations, endothelial dysfunction, and cardiovascular risk factors, independently from obesity, even if obesity aggravates the phenotype (7). Therefore, a timely diagnosis and proper management should be warranted.

Although much has been clarified in recent years on the pathogenesis, diagnosis, clinical manifestations, and therapy of PCOS, there are still many doubts and consequent uncertainties in the choice of the therapeutic approach in clinical practice.

To address some important concerns of PCOS and especially its possible treatment with nutraceuticals, a panel of Italian experts convened webinar on February 3, 2020, after an independent review of the literature. The panel included clinicians dealing with this condition such as endocrinologists and gynecologists. Six resolutions were proposed, debated, and agreed on after a thorough discussion. Then the resolutions were written, discussed in their final version, and approved in a final consensus webinar held on April 15.

The summary of the resolutions and the condensed expert opinions are reported in Table 2.

**Table 2:** Summary of resolutions and expert opinion.

	**Resolution**	**Condensed expert opinion**
1	PCOS is a well-established medical condition that negatively affects reproduction, general health, sexual health, and quality of life.	• PCOS is a multifaceted disease with an impact on various aspects of a woman's life, such as aesthetics, reproduction, metabolism, psychological well-being, and sexuality. • Phenotypization is fundamental for providing a tailored therapy.
2	The symptoms and signs of PCOS appear early in life especially in female newborns from PCOS carriers.	• Daughters of PCOS women inherit certain characteristics that become more evident across puberty. • Early recognition of PCOS in adolescence is fundamental to set up individualized strategies to ameliorate symptoms and to counteract reproductive and metabolic risks associated with this condition.
3	Women with PCOS have significantly increased risk of pregnancy-related complications including gestational diabetes.	• Women with PCOS have an increased risk of GDM than controls, especially if obesity/metabolic syndrome are present, and should be carefully investigated and monitored during early pregnancy with OGTT. • Changes in intestinal microbiota during pregnancy may contribute to the onset of metabolic dysfunction in both the mother and the offspring.
4	A male PCOS equivalent seems to exist, and it may impact on metabolic health and probably on reproduction.	• Male PCOS equivalent may be diagnosed in presence of PCOS-like hormonal pattern, metabolic abnormalities, overweight/obesity, and/or clinical signs of hyperandrogenism, above all in patients aged <35 years with a family history positive for PCOS. • The metabolic and hormonal profile should be assessed in first-degree male relatives of PCOS women and in men with early-onset AGA. This may help to prevent the risk of T2DM and CVD later in life. • Further studies are needed to confirm the existence of a male PCOS equivalent and to evaluate its impact on the testicular function.
5	The evidence supports that medical therapy for women with PCOS is effective, rational, and evidence-based.	• No single unified treatment for PCOS is available, and treatment should be individualized. • Targets for pharmacological treatment include biochemical and clinical androgen excess, menstrual irregularities, anovulation, insulin resistance, and metabolic profile. • Lifestyle counseling should be provided in all cases. • COCPs are the first-line treatment for long-term management of menstrual irregularities and hyperandrogenism. • Metformin should be recommended in overweight/obese adult PCOS women and considered in adolescents with PCOS for the management of weight, insulin resistance, and metabolic abnormalities.
6	The evidence supports a major research initiative to explore possible benefits of nutraceutical therapy for PCOS.	• MI and DCI show different insulin-mimetic properties. Inositol administration should be aimed to keep unaltered the MI/DCI ratio. • Treatment with MI/ALA combination may ameliorate hyperinsulinemia, decrease oxidative stress markers at oocyte level, and normalize endometrial inflammasome in PCOS women with idiopathic recurrent pregnancy loss. • The hormonal and clinical profile of overweight/obese women with PCOS may benefit from prolonged use of MI/ALA combination, such as a higher recovery of class II oocytes during ART. • NAFLD may be associated with PCOS. A timely diagnosis is warranted to avoid the NAFLD-related long-term complications. A nutraceutical approach could be useful in the treatment of NAFLD. • Hyperomocysteinemia may be associated with selected PCOS patients. Treatment with folic acid should be started to avoid the long-term consequences on the cardiovascular system. • Nutraceuticals, associated with diet and lifestyle modifications, can be important therapeutic option to manage pregnancy-related complications in PCOS pregnant patients.

*AGA, androgenic alopecia; ALA, α-lipoic acid; ART, assisted reproductive techniques; COCP, combined oral contraceptive pills; DCI, D-chiro-inositol; GDM, gestational diabetes mellitus; MI, myoinositol; NAFLD, non-alcoholic fatty liver disease; OGTT, oral glucose tolerance test; T2DM, type 2 diabetes mellitus*.

### Resolution 1: PCOS is a Well-Established Medical Condition That Negatively Affects Reproduction, General Health, Sexual Health, and Quality of Life

Polycystic ovary syndrome is the most frequent disorder in women of reproductive age, and it is also one of the most well-studied. Nevertheless, many unresolved questions regarding its pathogenesis, diagnosis, clinical manifestations, acute and chronic complications, and treatment are still debated (8). It is one of the most common causes of menstrual irregularities, such as amenorrhea, oligomenorrhea, and polymenorrhea, and is the primary cause of anovulatory infertility (9). Infertility is aggravated by a high percentage of miscarriages, as well as by low pregnancy and live birth rates after assisted reproductive technologies (ARTs) (5). In addition, women with PCOS tend to produce a greater number of oocytes during ART and to have higher rates of ovarian hyperstimulation syndrome and/or multiple gestations compared with non-PCOS infertile women (5). Furthermore, increased pregnancy complications and obstetric and neonatal risks are also associated with PCOS. These include gestational diabetes mellitus (GDM), preeclampsia, pregnancy-induced hypertension, postpartum hemorrhage and infection, preterm delivery, meconium aspiration, stillbirth, operative deliveries, and shoulder dystocia (5, 10, 11). The presence of obesity exacerbates all these complications and produces a blunted responsiveness to ovulation induction in proportion to its severity and, particularly, when the abdominal phenotype of obesity (visceral fat accumulation) is present (12–15).

The pathophysiological mechanisms by which PCOS negatively impacts on fertility are complex and not completely understood. Undoubtedly, hyperandrogenism, the consequent hyperestrogenemia, IR, and compensatory hyperinsulinemia play an important role acting on both the ovary and the endometrium (16–19). In addition, there is emerging evidence that proinflammatory cytokines and oxidative stress may directly impact on oocyte quality and may induce endothelial dysfunction, thus contributing to infertility (6, 12, 13). Some recent *ex vivo* and *in vivo* studies have also demonstrated that proinflammatory cytokines inhibit follicle-stimulating hormone (FSH) and luteinizing hormone (LH) receptor expression, thus impairing the regular follicular development and its luteinization. They also inhibit FSH-induced 17β-estradiol and progesterone secretion from granulosa cells obtained by preovulatory follicles and stimulate testosterone production from theca cells, all mechanisms that contribute to impair ovulation and, therefore, infertility (20–22).

Polycystic ovary syndrome is not only a reproductive endocrinopathy, but it is also a metabolic endocrinopathy, for the high prevalence of overweight/abdominal obesity, dyslipidemia, non-alcoholic fatty liver disease (NAFLD), type 2 diabetes mellitus (T2DM), and metabolic syndrome (MetS) (4, 23, 24). Obesity, hyperandrogenism, and IR are known risk factors for increasing NAFLD and T2DM occurrence in PCOS (20, 25, 26). Furthermore, these metabolic alterations are well-known additional risk factors for cardiovascular diseases (CVDs), although strong evidence in PCOS is lacking. Additionally, chronic low-grade inflammation is a risk factor for IR and T2DM (16, 27–29). In addition, many recent studies have clearly shown that PCOS is associated with anxiety (30–36) and depression (26–32, 37, 38), and with a poorer quality of life (27, 32, 34, 39–42), even in adolescence and young age (43). Both physical and mental distress may contribute to decrease the quality of life in PCOS (36, 44, 45) regardless of body mass index (BMI), hyperandrogenism, and socioeconomic status (33). However, the presence of obesity, menstrual disorders, clinical hyperandrogenism (acne, alopecia, hirsutism), and infertility may impact on the quality of life (26, 31, 34, 35, 46–49). Thus, infertility and hirsutism have been associated with both anxiety (26, 27, 50) and depression (29, 46) and alopecia with anxiety (23), whereas obesity and acne with depression (26, 31, 34). Accordingly, both weight loss and oral contraceptive use significantly improve several physical and mental domains related to quality of life, depressive symptoms, and anxiety disorders (51, 52). A high percentage of eating disorders, such as anorexia nervosa, bulimia nervosa, and binge eating disorder, have also been observed in PCOS (29, 53), particularly in the presence of anxiety and depression (49).

More contradictory results have been found regarding the association between PCOS and sexual dysfunctions, because some studies described a high prevalence of sexual dysfunctions (38, 46, 49, 54–56), whereas others did not (35, 57–64). The studies that have demonstrated a high prevalence (46, 50–53) described defect in arousal, poor lubrification (46) and pain during intercourse (52, 53), and high degree of sexual dissatisfaction (38, 50–52). All the aspects of sexual dysfunction described in PCOS are exacerbated by the presence of obesity (42, 50, 51, 61, 65), alopecia, and infertility, regardless of its duration (53). Contradictory results have been described regarding the association between sexual dysfunction and androgen circulating levels, with some studies demonstrating a positive correlation (50), others negative (61, 66), whereas others show no significant association (60). Hirsutism (38, 64), acne (67), and a sense of low attractiveness (38, 51) resulted mostly associated with low sexual satisfaction. As expected, PCOS women with the lowest sexual satisfaction exhibited an even stronger predilection toward anxiety and depression than the other women (34, 50, 51).

Altogether, these data demonstrate that PCOS is appropriately defined syndrome for the complexity and heterogeneity of the clinical manifestations, associated comorbidities, and clinical consequences that accompany the woman during reproductive and postreproductive age, thus impacting on the quality of life.

#### Expert Opinion

- Polycystic ovary syndrome is a multifaceted disease with an impact on various aspects of women's life, such as aesthetics, reproduction, metabolism, psychological well-being, and sexuality.
- The lean PCOS phenotype has a high risk of underdiagnosis and misdiagnosis with respect to the overweight/obese phenotype.
- The phenotypization of the woman with PCOS is fundamental for providing a tailored therapy, by taking advantage of the wide and diversified therapeutic availability.

### Resolution 2: The Symptoms and Signs of PCOS Appear Early in Life Especially in Female Newborns From PCOS Carriers

Nowadays, PCOS is a lifelong medical condition requiring a multispecialist vision for early diagnosis and an effective management and treatment plan over time (10). Preconception care, mainly in infertility clinics, is the golden moment to identify PCOS carriers because PCOS-like outcomes, especially in female offspring, result from both genetic and epigenetic mechanisms (68, 69). Indeed, specific PCOS-susceptibility loci may explain family predisposition and the variable clinical presentation of PCOS, including neuroendocrine, reproductive, and metabolic abnormalities (70). On the other hand, maternal–fetal interactions account for early signs of hyperandrogenism in offspring of PCOS carriers, resulting not only from heritability, but also from the intrauterine androgen excess of maternal and/or fetal origin, with the contribution of a dysfunctional placenta (71). Overexposure to androgens *in utero* influences the activity of multiple pathways regulating gonadotropin-releasing hormone (GnRH) pulsatility, follicular development, ovarian steroidogenesis, and insulin–glucose homeostasis (72).

Glucose intolerance and hyperinsulinemia in metabolically unhealthy PCOS mothers further contribute to the prenatal hyperandrogenic milieu, which plays a leading role in reprogramming female offspring for reproductive, behavioral, and metabolic PCOS-like traits with some gender differences (73). On the other hand, injuries to the fetoplacental unit deriving from gestational-related conditions, such as hypertension and/or T2DM, or unhealthy habits (smoking, lack of exercise, etc.) may have an influence on weight at birth reprogramming metabolic function even in offspring of mothers without PCOS (74).

During infancy and adolescence, this “metabolic memory” may likely induce a non-genetic inheritance of PCOS-like features, linked to overweight and/or abdominal obesity, which are still preventable with healthy habits (adequate diet, active lifestyle, etc.) (8, 75). Altogether, endocrine and metabolic events in intrauterine life become evident early postnatally (76) and may give origin to a high rate of overt signs and symptoms of PCOS manifesting at puberty and progressing with age (77). Apart from subtle signs of a compromised cardiometabolic health, predominantly in female offspring (76) the diagnosis of PCOS in mothers and/or elevated fetal testosterone has been also linked to pervasive developmental disabilities, autism spectrum disorder, and attention-deficit/hyperactivity disorder, an evidence recently confirmed in a US cohort of infants of PCOS mothers (78).

Polycystic ovary syndrome diagnosis during infancy and childhood is virtually impossible because symptoms are missing. However, the measurement of anti-Müllerian hormone (AMH), a surrogate marker of ovarian hyperandrogenism produced by small antral follicles (79), in daughters of women with PCOS may represent a reliable proxy. Indeed, AMH values are higher across puberty and correlate with LH and testosterone plasma levels, indicating multifollicular ovarian morphology resulting from enhanced recruitment, decreased depletion or even from follicular growing arrest, and reduced rate of atresia (18, 80). Newborns with low and high birth weights have higher AMH levels than normal birth weight infants tested at 2–3 months of age (81). However, body weight abnormalities at birth do not seem associated with having a PCOS mother (82). On the other hand, birth weight and thinness at birth independently predict symptoms of PCOS in adulthood (83). Many coexisting elements, such as maternal obesity, pregnancy complications, and other comorbidity make it difficult to identify the real contribution of PCOS mothers to health and development of offspring (84).

In any case, it is more likely that daughters of PCOS women have more abdominal fat distribution and signs of hyperandrogenism compared with control daughters across pubertal maturation (85). Increased glucose-stimulated insulin levels are also a consistent phenotype in the daughters of women with PCOS in mid to late puberty (86). Moreover, 5α-reductase, the key enzyme in androgen metabolism, catalyzing the irreversible conversion of testosterone to dihydrotestosterone (DHT) into the skin, is significantly increased during early childhood in daughters of women with PCOS (87). On the other hand, IR *per se* amplifies hyperandrogenic signs by enhancing the activity of 5α-reductase at the target levels (88).

Even though no causal relationship between excessive adiposity and early puberty has been clearly demonstrated, it is likely that these girls can accelerate their growth and maturation in a homeostatic attempt to reduce their abdominal obesity, showing a higher tendency to early pubarche and eventually early menarche (89). On the other hand, an excess of adipose tissue during adolescence undoubtedly increases the possibility of developing multifollicular ovarian morphology resembling PCOS-like aspect. This is also a sign of reproductive axis immaturity, and therefore, it is not considered a diagnostic criterion within 2 years after menarche. Irregular menses that persist 2 years after menarche may be a sign of PCOS, although they may continue up to the fifth year after menarche without developing PCOS. Circulating androgens reach adult levels generally by age 15 years, and hirsutism may increase progressively over the time (90). In adolescence, IR and hyperinsulinemia have a widespread impact on reproductive function by multiple mechanisms, including a synergic role with LH to stimulate the secretion of androgens from the ovarian theca. Moreover, insulin, by lowering liver sex hormone–binding globulin (SHBG) production, increases androgen bioavailability at target organs, further contributing to clinical signs of hyperandrogenism (91).

Finally, IR, independently from the extent of obesity and magnitude of androgen concentrations, may be present even in lean PCOS. However, transient hyperinsulinemia is typical at puberty and may amplify the individual predisposition to develop PCOS (92).

Collectively, these data indicate that clinical signs of hyperandrogenism observed in adolescent PCOS, in particular acne, are likely influenced not only by the abundance of DHT at the level of the pilosebaceous unit, but also by different abnormal insulin signaling in concert with nutritional aspects (93). Symptoms of androgen excess may be quite common in adolescents and may cause significant distress well before a final diagnosis of PCOS can be formulated. To ameliorate hyperandrogenic signs, it is essential to find therapeutic strategies to fill the gap between the physiological changes of puberty and the clear clinical picture that becomes evident some years following menarche (94). Therefore, knowing that an intergenerational risk for PCOS exists serves to prevent its onset from intrauterine to adult life.

#### Expert Opinion

- Polycystic ovary syndrome is a clear example of transgenerational disease that results from genetic and epigenetic mechanisms from prenatal life to adulthood.
- Daughters of women with PCOS inherit certain characteristics that become more evident across puberty. These include abdominal fat distribution and signs of hyperandrogenism.
- Early recognition of PCOS in adolescence is fundamental to set up individualized strategies to ameliorate symptoms and to counteract reproductive and metabolic risks associated with this condition.

### Resolution 3: Women With PCOS Have Significantly Increased Risk of Pregnancy-Related Complications Including Gestational Diabetes

Polycystic ovary syndrome is a primary risk factor for adverse pregnancy outcomes. A meta-analysis conducted by Kjerulff et al. (95) indicated that pregnancy in PCOS patients is associated with increased risk of GDM, pregnancy-induced hypertension, preeclampsia, preterm delivery, and small-for-gestational age, and according to various data, the risk of miscarriage in PCOS women is reported to be three times higher than in healthy women (96). Indeed, PCOS could be included as a risk factor for GDM (97).

During pregnancy, a physiologic insulin insensitivity occurs because of the release of placental hormones. These hormones promote nutrient utilization by the fetus, but on the other hand, IR associated with pregnancy is the main pathogenic mechanism leading the development of GDM in predisposed women such as PCOS. All lipoprotein subclasses and lipids are markedly increased in pregnant women, and the most pronounced differences are observed for the intermediate-density, low-density (LDL), and high-density lipoprotein (HDL) triglyceride concentrations (98). All these metabolic alterations lead to an increase in the plasma concentration of circulating proinflammatory cytokines, such as tumor necrosis factor α and interleukin 6 (IL-6), with reduction of plasma anti-inflammatory molecule levels such as adiponectin and IL-10 (99). Furthermore, inflammatory mediator overexpression, together with an increase of reactive oxygen species (ROS), could lead to metabolic alterations and vascular disease and induce inhibition of the insulin signaling pathway, thus resulting in IR, reduced insulin gene expression, and, consequently, reduced β-cell insulin secretion and GDM (100). In many cases, these factors are already present in women with PCOS, and therefore pregnancy can be the final hit in the onset of DM.

According to the current Italian guidelines on GDM, PCOS women carrying high risk (i.e., obesity, previous macrosomia or GDM, fasting blood sugar 100–125 mg/dL at the beginning of pregnancy) should be investigated by oral glucose tolerance test (OGTT) between the 16th and 18th week of gestation and, if normal, by repeating it between the 24th and 28th week of pregnancy (101).

A crucial role of the intestinal microbiota in pregnancy-related GDM has been suggested. In the first trimester, the composition of the gut microbiota is similar to that of a non-pregnant woman. In the following months, the variability of microorganisms decreases, and there is an increase in the populations of *Proteobacteria* and *Actinobacteria*; *Bifidobacteria* belong to the latter and play a pivotal role in the defense against pathogenic bacteria, in strengthening the intestinal barrier, and in the nutrients' metabolism. The intestine of a pregnant woman can become more permeable favoring the so-called “bacterial translocation” so that the fetus can come into contact with microorganisms of maternal origin (microbes have been found in the blood of the umbilical cord, in the amniotic fluid, and even in the meconium) (102–104). Furthermore, according to the most widespread theories, the baby's intestine is colonized during childbirth by the bacteria of the maternal microbiota. If the pregnant woman is in dysbiosis, the fetus will not receive bifid but other bacterial strains, which could lead to a greater exposure of the newborn to diseases. This imbalance can also lead to a greater absorption of calories with a consequent weight increase of the pregnant woman and a greater risk of developing GDM. Even the newborn could more easily develop childhood diabetes, allergies, and childhood obesity (102–104).

#### Expert Opinion

- Women with PCOS have an increased risk of GDM than controls, especially if obesity/MetS are present, and should be carefully investigated and monitored during early pregnancy with OGTT.
- Changes in intestinal microbiota during pregnancy may contribute to the onset of metabolic dysfunction in both the mother and the offspring.

### Resolution 4: A Male PCOS Equivalent Seems to Exist, and it May Impact on Metabolic Health and Probably on Reproduction

The genetic background of PCOS (105) suggests the existence of a male PCOS equivalent (106). Indeed, even male relatives inherit the same genetic factors predisposing to female PCOS. However, little is known about the putative presence of a male-PCOS and its possible consequences in men.

Kinship of PCOS women has an increased risk to develop metabolic abnormalities and CVDs, independently of the gender. Indeed, their siblings have a higher prevalence of IR, hyperinsulinemia, dyslipidemia, and hypertension already at a young age (<40 years) (107–109). Young first-degree male relatives have endothelial dysfunction that is not present in female PCOS patients (110). Therefore, the male relatives have an increased metabolic and cardiovascular risk. Furthermore, obesity, MetS, T2DM, and CVDs are more frequently diagnosed in male and female first-degree relatives of PCOS women compared to those with a negative PCOS family history (107, 108).

Only few studies have explored hormonal abnormalities in the first-degree male relatives of women with PCOS. They have been shown to have higher dehydroepiandrosterone (DHEAS) level (111, 112), which suggests the presence of a similar steroidogenic abnormality reported in their sisters. In addition, they have higher levels of AMH, LH, and FSH compared with controls (113). Moreover, a higher LH and FSH response to GnRH stimulation test has been reported in these men compared to controls (114), suggesting the presence of an abnormal GnRH-induced gonadotropin release.

The presence of metabolic, cardiovascular, and hormonal alterations in the male relative of women with PCOS supports the existence of a male PCOS equivalent. Accordingly, several authors have shown the occurrence of early-onset (<35 years) androgenetic alopecia (AGA) [grade ≥III, Hamilton–Norwood scale (115, 116)] in male relatives of PCOS women (117, 118). Therefore, early-onset AGA has been proposed as a clinical sign of the male PCOS equivalent. On this account, the hormonal and metabolic pattern has been evaluated in men with early-onset AGA, independently from the kinship with PCOS women. A meta-analysis carried out in 1,009 unrelated men showed increased LH and DHEAS, decreased SHBG, a downward trend for FSH, and an upward trend for the LH/FSH ratio in patients with early-onset AGA compared with controls (119), therefore resembling the female PCOS hormonal pattern. The same meta-analysis found a significant increase of insulin levels and Homeostatic Model Assessment (HOMA) index, total and LDL cholesterol, and triglycerides in patients with respect to controls, already before the age of 35 years. Unfortunately, there are no data on the reproductive function in men with early-onset AGA. Although it is plausible that the reproductive potential of subjects with “male PCOS phenotype” might be compromised similarly to women with PCOS, one could also speculate that fertility could be even improved in these subjects, highlighting therefore an intriguing evolutionary paradox.

By contrast, a large amount of data has been produced on the metabolic, cardiovascular, and prostatic sequelae in aging men with early-onset AGA. A hospital-based analysis reported a higher risk for myocardial infarction in 665 patients with respect to 772 controls; the risk was higher for severe vertex [Relative risk(RR), 3.4] compared with frontal AGA (RR, 0.9) (120). A retrospective study carried out in 22,071 men aged 40–84 years found an increased prevalence of T2DM in patients with severe vertex AGA than those belonging to other hair-pattern categories (121). Moreover, the Framingham Study showed a positive correlation between AGA progression and coronary heart disease (CHD) (122). The NHANES I Epidemiologic Follow-up Study (3,932 men aged 25–73 years) reported that severe AGA was positively associated with mortality for CHD in men before 55 years of age (123). Furthermore, a meta-analysis on 29,254 participants confirmed the positive correlation between AGA and CHD, IR, hyperinsulinemia, and MetS in both genders (124). The level of the evidence is high enough that AGA has been already proposed as an independent predictor of mortality for T2DM and CVDs (125). Finally, prostate inflammation, hyperplasia, and even cancer have been reported in men with early-onset AGA (120). Current evidence supports a relationship between prostate inflammation and hyperplasia with IR and hyperinsulinemia, whose mechanisms are not clear yet. It might be speculated that prostate diseases represent long-term consequence of male PCOS equivalent, whose pathogenesis includes metabolic abnormalities.

In conclusion, PCOS syndrome is not simply a primarily ovarian disorder, because the core of its pathogenesis is a metabolic dysfunction, as also suggested by the long-term complications found in these patients. The genetic predisposition to develop a syndrome with a negative impact on metabolism can occur also in men. Current evidence indicates the existence of metabolic, cardiovascular, and hormonal abnormalities in the first-degree males of PCOS women. The higher prevalence of early-onset AGA found in these men supports that it may represent a phenotypic feature of the male PCOS equivalent. Accordingly, patients with early-onset AGA show a PCOS-like hormonal pattern, increased insulin, HOMA index, total and LDL cholesterol, and triglycerides compared to age-matched controls. Similar to female PCOS, long-term consequences of early-onset AGA (male PCOS equivalent) include T2DM, CVDs, and a higher mortality for CHD. Hence, the male PCOS syndrome might be defined as an endocrine syndrome with a metabolic background predisposing to the development of T2DM, CVDs, and prostatic diseases later in life.

#### Expert Opinion

- Male PCOS equivalent may be diagnosed in presence of PCOS-like hormonal pattern (increased DHEAS, AMH, LH, LH/FSH ratio, high calculated free testosterone), metabolic abnormalities (IR, hyperinsulinemia, low SHBG levels, hyperglycemia), overweight/obesity, and/or clinical signs of hyperandrogenism (mainly early-onset AGA), above all in patients younger than 35 years with a family history positive for PCOS.
- The metabolic and hormonal profile should be assessed in first-degree male relatives of PCOS women and in men with early-onset AGA. This may help to prevent the risk of T2DM and CVD later in life.
- Further studies are needed to confirm the existence of a male PCOS equivalent and to evaluate its impact on the testicular function.

### Resolution 5: Evidence Supports That Medical Therapy for Women With PCOS is Effective, Rational, and Evidence-Based

Pharmacological treatments commonly used in women with PCOS are off-label because neither the Food and Drug Administration nor the European Medicines Agency has ever approved a specific drug for PCOS treatment. However, they are widely used and their efficacy is evidence-based and rational (74).

Therapeutic approaches aim to improve disease-related symptoms and should therefore target hyperandrogenism, the consequences of ovarian dysfunction (menstrual irregularities, infertility), and/or the associated metabolic disorders. Apart from ovulation induction and treatment of infertility and topical-aesthetic therapies for hirsutism and acne, pharmacological treatment is recommended when the first-line approach represented by lifestyle modifications (diet and/or physical activity) is not able alone to ameliorate the symptoms and signs of PCOS. The evidence for medical therapy of PCOS is summarized in a recent report from 37 societies and organizations that published the first international evidence-based guideline for the assessment and management of PCOS (94).

Combined oral contraceptive pills (COCPs) are first-line pharmacological management for menstrual irregularity and hyperandrogenism, whereas metformin (alone or in addition to COCPs) is recommended for the management of metabolic alterations associated with PCOS (52, 126). The rational and efficacy of COCPs treatment are linked to their ability to decrease the pulse frequency of GnRH (and therefore the secretion of FSH and LH), the ability of progestin combined to lack of estrogen positive feedback to suppress midcycle LH surge and LH levels (and thus ovarian androgen production), and to the ability of different estrogen-progestin combinations to increase SHBG, thereby reducing bioavailable free androgens. Also, some progestins have antiandrogenic properties, because of their antagonizing effects on the androgen receptor and to the inhibition of 5α-reductase activity.

Combined oral contraceptive pills exert a class effect on PCOS. However, in order to reduce the thromboembolic risk, the lowest effective estrogen doses (such as 20–30 μg of ethinylestradiol) or the use of natural estradiol should be preferred. Even though clinical trials (127) almost always use COCPs containing antiandrogen progestins (due to higher effect on hyperandrogenism symptoms), the guidelines do not recommend a specific progestin. A recent meta-analysis showed that COCPs containing cyproterone acetate are more effective in suppressing gonadotropins, leading to a decrease in androgen levels (128). Another meta-analysis showed that COCPs containing drospirenone have a more potent effect than COCPs containing chlormadinone acetate in the reduction of circulating androgen-related hirsutism (129). A risk–benefit assessment should take place when selecting a specific COCPs in the individual woman to minimize potential metabolic and thromboembolic consequences. Indeed, third-generation and antiandrogenic progestins are the drugs of choice for hirsutism, alopecia, and acne, but may carry higher risks as compared to the second generation. In combination with COCPs, antiandrogens—considering their potential hepatotoxicity and other side effects—should only be considered in PCOS when androgen-related alopecia and/or hirsutism have to be treated, after 6 months or more failure of COCPs and cosmetic therapy (130). Antiandrogens must be used with effective contraception, to avoid male fetal undervirilization.

Metformin, in addition to lifestyle changes, should be recommended in adult women with BMI ≥25 kg/m^2^ and considered in adolescents with PCOS for the management of weight (131) and metabolic abnormalities. It is most beneficial in high metabolic risk groups including patients with T2DM risk factors or impaired glucose tolerance and non-obese IR PCOS women; it should be started at a low dose (with 500-mg increments 1–2 weekly), using extended-release preparations, to minimize gastrointestinal side effects. Conclusive data suggest that metformin alone has benefits for adult women for management of weight, hormonal (testosterone), and metabolic outcomes (fasting glucose and insulin, LDL cholesterol), especially for women with BMI ≥25 kg/m^2^, whereas COCPs are more effective than metformin for menstrual regulation, and metformin combined with COCPs may be useful for the management of metabolic features (95). Finally, there is reliable evidence regarding the use of metformin for anthropometric (reduction of body weight and decrease of waist) outcomes and COCPs for hyperandrogenism in women with PCOS (132). In fact, in overweight women with PCOS, metformin plus lifestyle changes might be the best intervention to improve IR and total triglycerides, whereas COCPs plus lifestyle changes appear to be the best intervention for the reduction of total cholesterol and LDL cholesterol, therefore suggesting a combined treatment with metformin and COCPs in these patients (133).

A recent meta-analysis (134) suggested that orlistat, an antiobesity drug that decreases fat absorption from the intestine, is more effective than metformin in weight loss, LDL and cholesterol level, and IR. Another meta-analysis aimed at evaluating the efficacy and safety of glucagon-like peptide 1 (GLP-1) receptor agonists showed that GLP-1 receptor agonists were more effective than metformin in improving insulin sensitivity and reducing BMI, suggesting that GLP-1 receptor agonists might be a good choice for obese patients with PCOS, especially those with IR (135). However, for both orlistat and GLP-1 receptor agonists, the available evidence is of low quality and therefore inconclusive.

#### Expert Opinion

- No single unified treatment for PCOS is available. Treatment should be individualized and adapted to the patient. Treatment should aim to improve those symptoms and signs that represent the patient's real needs and can be changed over time.
- Targets for pharmacological treatment include biochemical and clinical androgen excess, menstrual irregularities, anovulation, insulin resistance, and metabolic profile.
- Lifestyle counseling should be provided in all cases given the deleterious effects of abdominal adiposity and obesity on the cardiometabolic risk profile.
- Combined oral contraceptive pills should be used as a first-line treatment for long-term management of menstrual irregularities and hyperandrogenism.
- No specific COCPs are recommended. However, those containing the lowest effective doses of estrogens (20–30 μg ethinylestradiol) or preparations with natural estrogens should be preferred.
- Combined oral contraceptive pills containing an antiandrogenic progestin are the drugs of choice for hirsutism, alopecia, and acne.
- Metformin should be recommended in overweight/obese adult PCOS women and considered in adolescents and lean IR PCOS women for the management of weight, IR, and metabolic abnormalities.

### Resolution 6: Evidence Supporting the Possible Benefits of Nutraceutical Therapy in PCOS

Several nutraceuticals have been investigated for their possible benefits in the management of PCOS-related IR, anovulation, liver inflammation and hyperhomocysteinemia. They include inositols, α-lipoic acid (ALA), silybin, resveratrol, vitamin D, vitamin E, and folic acid, whose evidences are summarized in the following resolution.

Myoinositol (MI) and d-chiro-inositol (DCI) are both implicated in the modulation of insulin signaling on steroid and ovarian folliculogenesis. In recent years, several studies have shown their effectiveness in patients with PCOS (136). The choice of the inositol to be prescribed to women with PCOS should be guided as much as possible by an evidence-based background. Both MI and DCI show insulin-mimetic properties and decrease postprandial blood glucose, but display a different peripheral action (137). Particularly, DCI acts on glycogen synthesis at the level of the skeletal muscle (138) by up-regulating GLUT4 expression (130). Myoinositol is involved in glucose uptake and FSH signaling in the ovary, whereas DCI influences the insulin-dependent synthesis of androgens (139). Epimerase converts MI into DCI in an insulin-sensitive manner and IR dramatically reduces the amount of epimerization (140) leading to a deficiency of DCI-dependent insulin-sensitive properties in PCOS patients. In human ovaries, ~99% of the intracellular pool of inositol is constituted of MI, and the remaining part of DCI (132, 141). An imbalance in the ovarian concentration between MI and DCI can compromise the FSH pulsatility rate so that we highlight the importance of keeping unaltered the MI/DCI ratio, rather than restoring only one of the two inositols (142, 143).

Recent therapeutic evidence addressed the importance of the association between ALA and MI or DCI. α-Lipoic acid is a powerful antioxidant and enzymatic cofactor of the mitochondrial respiratory chain, in turn, capable of increasing insulin sensitivity. It is believed to directly scavenge ROS and reactive nitrogen species (RNS), both *in vitro* and *in vivo*. α-Lipoic acid regenerates essential antioxidant molecules, that is, coenzyme Q10, vitamin C, vitamin E, and chelates several heavy metals involved in oxidative processes. Also, ALA can repair oxidative stress-damaged proteins, lipids, and DNA (144). Also, it is supposed to improve glycemic control due to the ALA-induced GLUT-4 expression with subsequent uptake of glucose within tissues (145). Also, systematic review and meta-analysis suggested that ALA might be able to decrease serum leptin (146) concentrations especially in younger adults depending on the longer time of assumption; also, a significant increase in serum levels of adiponectin in studies, which lasted for more than 8 weeks (146). α-Lipoic acid possibly decreases both adipose tissue leptin and circulating leptin mRNA levels through enhanced peroxisome proliferator-activated receptor-γ activity, which has an important role in decreasing leptin gene expression and in determining appropriate insulin signaling (147). This effect may be considered important given a recent meta-analysis suggesting that ALA decreased body weight among participants with obesity; therefore, it might be possible that ALA by AMPK activation and weight reduction decreases leptin and increases adiponectin levels (146, 148), thus representing a very promising molecule in reducing some frequent features of PCOS, that is, increased body weight and inflammation (145).

Several studies support the use of ALA (149, 150) for its direct and indirect antioxidant effects through the regeneration of other antioxidants and its anti-inflammatory activities exerted by inhibiting some cytokines. Some authors have shown that the endometrial inflammasome, in which prevails the overexpression/activation of NALP-3, is associated with idiopathic recurrent pregnancy loss (RPL) and that the combined treatment can re-modulate the endometrial pathway of NALP-3 with a decrease of the concentrations of apoptotic cytokines (151). In a group of PCOS patients, treatment with ALA and DCI led to a non-significant improvement of clinical and metabolic features, that is, insulin, BMI, HDL, and menstrual cyclicity, compared to an untreated control group (152). Masharani and colleagues highlighted the benefits of ALA on glucose uptake in lean PCOS patients, although this evidence is limited by the poor number of patients treated (153). In the pilot cohort study by De Cicco and colleagues, 40 patients with normoinsulinemic overweight PCOS were treated for 6 months with the MI plus ALA combination and the results showed a decrease in BMI, waist-hip ratio, hirsutism score, AMH, ovarian volume, and antral follicle count, and an increase in the number of menstrual cycles (from 2 to 5) (154). Genazzani and colleagues demonstrated that the combined administration of ALA and MI to obese PCOS women, grouped by the presence or absence of familiarity with type 1 or T2DM, led to an improvement of insulin sensitivity in patients with T2DM familiarity and an improvement in the insulin response to OGTT in both groups, unlike what was observed after treatment with MI alone (155).

In PCOS women with familiarity for T2DM and therefore with a reduced expression of lipoic acid synthase and epimerase, the combination of ALA plus MI markedly reactivated the stimulus on GLUT4 and therefore was able to improve insulin sensitivity (156). Also, the treatment with ALA (400 mg/d) improved the metabolic features especially in the presence of T2DM. Numerous evidence show that the treatment with MI and/or DCI improves reproductive outcomes in terms of spontaneous induction of ovulation and leads to a modification of the standard treatments used for ART, that is, a reduction in either the gonadotropin units required for the controlled ovarian hyperstimulation (COH) or in the overall number of days required to reach the maximal stimulation (157). At the same time, an improvement in oocyte quality and pregnancy rate has been observed both in humans (158–161) and in animal models (162). A randomized trial evaluated the effects of MI alone or in combination with ALA in a population of non-obese PCOS women undergoing ART. Significant reductions in BMI, insulin (baseline and after OGTT), ovarian volume, and gonadotropin units used for COH were observed. The oocyte quality was inversely correlated with the decrease of the BMI, resulting in a greater recovery of M-II oocytes, the formation of classes I and II embryos, and the increase in the pregnancy rate (163).

Evidence has shown the role of nutraceuticals in treating NAFLD. Silybin seems effective to treat PCOS-related NAFLD, which is defined as the clustering of triglycerides in macro- or micro-vesicles in more than 5% of hepatocytes. NAFLD is frequently present in PCOS women, with a diagnosis rate of up to 40% in the lean phenotype (164) and nearly 50% of obese PCOS women (165). It may develop into cirrhosis and hepatic failure, and therefore, its identification and treatment are of importance in patients with PCOS. A large body of *in vitro* evidence supports the direct benefit of incubation with silybin on hepatocytes in NAFLD models, mainly due to its capacity to reverse lipid accumulation in the liver (166–168). A controlled study in 90 NAFLD patients and 30 healthy controls reported the effectiveness of 6-month administration of silybin, vitamin D, and vitamin E on NAFLD fibrosis score, metabolic markers, oxidative stress, and endothelial dysfunction (169). Finally, the use of the polyphenol resveratrol is associated with the reduction of circulating tryglicerides (170). Altogether these preliminary reports suggest that the role of nutraceuticals in the treatment of PCOS may be extended to NAFLD, though focused randomized controlled trials (RCTs) need to be accomplished.

Hyperhomocysteinemia occurs in some patients with PCOS, in particular in those with a greater metabolic derangement (women with IR or hyperinsulinemia) (171). A genomic study evaluated the effect of the G2706A and G1051A polymorphisms of the *ABCA1* gene on homocysteine serum levels in a cohort of 98 PCOS patients and 93 healthy controls. Significantly higher homocysteine levels were found in PCOS patients with the *ABCA* G1051A mutant genotype than heterozygotes or wild type (172). Interestingly, among patients with PCOS, homocysteine levels may predict pregnancy outcome, being higher in PCOS patients with recurrent pregnancy loss compared with fertile PCOS controls (173). Hyperhomocysteinemia predicts the risk of developing atherosclerosis in patients with MetS (174) and it is a risk factor for ischemic heart disease and stroke (175). A meta-analysis has shown that folic acid lowers the risk of stroke by 10% and of overall CVD by 4% in patients with hyperomocysteinemia (176). This evidence justify the assessment of homocysteine and its possible treatment in PCOS patients.

Diet and healthy lifestyles are universally recognized treatments for obesity and IR before and during pregnancy in PCOS women (177). Remarkably, in most countries, none of the oral hypoglycemic agents are allowed during the gestational period and insulin remains the only therapeutic option to manage GDM. However, in the last years, in a variety of experimental models, inositol and antioxidant supplementation have shown insulin-sensitizing, anti-inflammatory, and antioxidant properties, providing an important therapeutic opportunity for women with PCOS and GDM (178, 179). On the other hand, combined inositol treatment improves blood pressure, glucose, and leptin levels in pregnant women with metabolic-like syndrome phenotype (180). Promising data from RCTs of MI dietary supplementation have shown positive results in terms of ameliorating IR, the incidence of GDM, and its adverse outcomes (181). Interesting studies report that MI can prevent the development of GDM by improving glucose metabolism in PCOS patients (126, 182). In this condition, a diet rich in fat-soluble vitamins, fiber, and antioxidants may play a positive role (183). For example, chronic consumption of quercetin, a flavonoid antioxidant, seems to alleviate fasting and postprandial hyperglycemia in animal models of DM, in part by inhibiting α-glucosidase activity (184). α-Lipoic acid as a supplemental agent has recently been proposed in T2DM, obesity, and pregnancies complicated by GDM (156). α-Lipoic acid can decrease either glycemic or inflammation levels especially in dysmetabolic patients with an increased T2DM risk occurrence (144). A recent meta-analysis indicated that it decreases body weight and leptin levels and increases adiponectin levels in obese participants (146). Other antioxidants, such as epigallocatechin 3-gallate, vitamin E, and resveratrol, have been suggested to have similar effects (170). Vitamin D replacement may have some beneficial effects on IR (185).

Finally, a meta-analysis of 11 RCTs including 719 pregnant women with GDM recently analyzed the effect of a 4- to 8-week-long probiotic administration on pregnancy outcome, glycemic control, blood lipid profile, inflammation, and oxidative stress. *Lactobacillus* and *Bifidobacterium* were prescribed in almost all RCTs. Probiotic administration resulted in a lower risk of offspring's hyperbilirubinemia. The authors also found the significant improvement of maternal glycemia, lipid profile, inflammation, and oxidative stress (186). In conclusion, inositols, ALA, vitamins, antioxidant supplementation, and multiple combinations of these compounds, associated with diet and lifestyle modifications, can be an important therapeutic option to manage PCOS and pregnancy-related complications. Evaluation of the safety of these compounds and their combinations concludes that they are safe, and there is no evidence of adverse events both in mothers and fetuses (187).

#### Expert Opinion

- Myoinositol and DCI show different insulin-mimetic properties. Inositol administration should be aimed to keep unaltered the MI/DCI ratio.
- Treatment with MI/ALA combination may ameliorate hyperinsulinemia, decrease oxidative stress markers at oocyte level, and normalize endometrial inflammasome in PCOS women with idiopathic recurrent pregnancy loss.
- The hormonal and clinical profile of overweight/obese women with PCOS may benefit from prolonged use of MI/ALA combination, such as a higher recovery of class II oocytes during ART.
- Non-alcoholic fatty liver disease may be associated with PCOS. A timely diagnosis is warranted to avoid the NAFLD-related long-term complications. A nutraceutical approach could be useful in the treatment of NAFLD.
- Hyperhomocysteinemia may be associated with selected PCOS patients. Treatment with folic acid should be started to avoid the long-term consequences on the cardiovascular system.
- Nutraceuticals, associated with diet and lifestyle modifications, can be important therapeutic option to manage pregnancy-related complications in PCOS pregnant patients.
- Gut microbiota integrity is important to prevent pregnancy-related complications in PCOS women.

## Discussion

Polycystic ovary syndrome is a clinically heterogeneous syndrome. Given the wide range of available therapeutic choices, it is important to recognize the exact phenotype (8) to provide the best evidence-based approach. Fetal exposure to a hyperinsulinemic and hyperandrogenic uterine environment leads to epigenetic changes (82) that, in addition to the genetic background, confer the susceptibility of developing a metabolic derangement in the offspring (male and female) of PCOS women. This represents the rational basis to look for PCOS early in life, allowing a timely diagnosis already in adolescence. Women with PCOS are exposed to an increased metabolic (and, probably, cardiovascular) risk later in life (7). Also, still in the youth, PCOS women have a higher risk of developing GDM compared to their counterparts, and therefore, proper counseling has to be made in those women who wish to become pregnant (97). Interestingly, the evidence pointed out to the possible existence of a male PCOS equivalent (106), which, similarly to the classic female phenotype (119), deserves to be early detected to avoid its long-term cardiometabolic (125) and, possibly, reproductive complications.

Several therapeutic choices are currently available for the management of PCOS women. The assessment of anthropometric data, biochemical and clinical androgen excess, menstrual irregularities, oligoanovulation, IR, and metabolic profile are required to adopt the best effective therapeutic strategy (52). Therapeutic options range from lifestyle changes (48) and aesthetic interventions to nutraceuticals and, finally, medications. Lifestyle counseling is required for a proper PCOS management in all patients. Although no specific COCP is recommended, this document suggests a prescription of the lowest effective estrogen doses (74). Those containing neutral/antiandrogenic progestin or antiandrogens should be preferred in the case of hirsutism, alopecia, and acne (130). Metformin should be recommended in overweight/obese adult PCOS women and considered in adolescents with PCOS for the management of weight, IR, and metabolic abnormalities. Furthermore, metformin may be an option to improve fertility, especially in IR overweight PCOS women.

Nutraceutical therapy seems to represent a challenge for the treatment of IR-PCOS women, with particular attention to restoring the MI/DCI ratio, which is unbalanced because of the insulin-dependent epimerase dysregulation, especially in obese patients (140), and possible usefulness of ALA. Many other supplemental elements are also available for the treatment of additional PCOS features, such as NAFLD or hyperomocysteinemia. However, further unbiased RCTs should be warranted to provide evidence-based data on the correct use of nutraceutical and appropriate timing in different phenotypes of PCOS patients.

This expert panel strongly encourages well-performed RCTs focused on analyzing the uncovered issues related to the application of nutraceuticals in these patients.

## Author Contributions

All authors contributed to conceptualization, methodology, bibliographic search and literature review, original draft preparation, review, editing, and have read and agreed to the published version of the manuscript.

## Conflict of Interest

The authors declare that the research was conducted in the absence of any commercial or financial relationships that could be construed as a potential conflict of interest. The handling editor declared a past co-authorship with the authors RR, AC, and AF.
